# Effects of Lactic Acid Bacteria-Directed Screening on Flavor and Functional Properties of Fermented Corn Protein Hydrolysate

**DOI:** 10.3390/foods14173074

**Published:** 2025-08-31

**Authors:** Shanzi Cong, Meng Sun, Yujia Cao, Hongji Zhao, Jingyi Sun, Guanlong Li, Xiaolan Liu, Nan Hu

**Affiliations:** Heilongjiang Provincial Key Laboratory of Corn Deep Processing Theory and Technology, College of Food and Bioengineering, Qiqihar University, Qiqihar 161006, China

**Keywords:** corn protein, *Limosilactobacillus*, fermentation, volatile compounds, antioxidant, processing characteristics

## Abstract

This study aims to screen out high-yield protease lactic acid bacteria (LAB) from cheese and analyze the flavor and functional characteristics of their fermentation of corn protein hydrolysate (CPH). *Lacticaseibacillus rhamnosus* ZYN-71 and *Limosilactobacillus fermentum* ZYN-76 were isolated and screened by traditional biological methods. Then, the two strains synergistically fermented CPH, and it was found that the scavenging rate of DPPH, ·OH, and O^2−^· and the chelating ability of Fe^2+^ of the fermented CPH increased by 22.85%, 3.82%, 63.37%, and 43.27%, respectively. Meanwhile, the solubility, water-holding capacity, oil-holding capacity, foaming property, foam stability, emulsification property, and emulsification stability had also been improved to varying degrees. The aroma of the CPH after fermentation mainly consisted of aldehydes (20.2%) and nitrogen heterocyclic compounds (19.4%), and the content of off-flavor components was reduced. LAB fermentation effectively improves the practical problems existing in the current application of corn proteolytic products. This research can provide a research basis for corn protein-related products.

## 1. Introduction

Corn gluten meal (CGM), a key by-product from wet-milled corn starch production and brewing purification, is projected to reach 1.63 million tons in China by 2024. Composed primarily of gliadin (68% corn protein and 28% gluten) with poor water solubility, CGM significantly limits its food industry applications [1]. Current processing methods aim to enhance its functionality by modifying the protein’s molecular structure through various techniques.

CPH is a mixture of oligopeptides obtained through enzymatic hydrolysis of CGM [2]. CPH exhibits excellent processing properties and diverse physiological activities, including immune regulation, alcohol metabolism support, liver protection, hypertension resistance, antioxidant effects, and anti-fatigue functions [3,4]. Liu et al. [5] developed CPH using composite enzyme hydrolysis of CGM, revealing its enhanced stability and antioxidant activity. The identification of antioxidant cyclic peptides from this process holds significant implications for functional food development. Nurhartadi et al. [6] demonstrated that CPH derived from CGM hydrolyzed with pepsin exhibits potent antibacterial activity against *Staphylococcus aureus*, making it a novel antimicrobial agent for controlling foodborne *Staphylococcus aureus*. Compared to CGM, CPH demonstrates improved processing characteristics and nutritional value. However, the preparation process exposes numerous hydrophobic amino acid residues, resulting in pronounced bitterness and off-flavors that limit its application scope [7].

In recent years, with growing health awareness, public interest in gut health and LAB has significantly increased. Most LABs (*Lactiplantibacillus plantarum*, *Lacticaseibacillus rhamnosus*, *Lacticaseibacillus paracasei*, etc.) are recognized as safe microorganisms by the generally recognized as safe (GRAS) standard and widely used in food processing. When fermented by LABs, raw materials gain enhanced functional properties, antioxidant capabilities, and distinctive flavors and textures [8]. Han et al. [9] demonstrated that *Lacticaseibacillus casei*, *Lacticaseibacillus paracasei*, or *Lactiplantibacillus planturum* fermentation can alter the tertiary structure of plant proteins, promoting interactions between extracellular polysaccharides and proteins to form dense gel networks. This process improves soy protein’s gel characteristics, enhances digestibility, and boosts water retention and oil stability. EI Yourself et al. [10] found that the co-fermentation of *Lactobacillus plantarum* with peas eliminates odor-causing compounds like aldehydes, ketones, and furans while producing ester compounds with fruity and floral aromas, thereby improving overall flavor quality. These findings indicate that LAB applications in protein-based food ingredients effectively enhance product quality and mitigate undesirable flavors.

Recently, Cong et al. [11] identified LABs from blueberry peels that exhibit strong fermentation characteristics, antioxidant properties, and probiotic capabilities. As blueberries are highly acidic fruits, LABs selected through directed screening on their peels demonstrate excellent fermentation capacity for acidic fruits and vegetables. It is believed that targeted selection of these LAB will yield strains suitable for CPH applications while addressing current technical challenges. Therefore, this study employed both traditional biological methods and modern molecular biology techniques to isolate and screen high-proteinase-producing LAB strains from traditionally fermented cheese. The research further analyzed the effects of LAB fermentation on the flavor, antioxidant activity, and processing characteristics of CPH. These findings aim to provide a research foundation for the processing and application of CPH.

## 2. Materials and Methods

### 2.1. Isolation and Purification of LAB in Cheese

The cheese was sourced from the Zhalantun City Farmers’ Market. The isolation and purification of LAB followed the method described by Cong et al. [12]. A 10 g cheese sample was thoroughly mixed with 90 mL sterile water, incubated at 37 °C for 48 h to prepare a bacterial suspension. After diluting the suspension in a 10-fold gradient, 100 μL of the diluted solution was spread onto a 2% (*w*/*w*) CaCO_3_ de Man-Rogosa-Sharpe (MRS) solid medium (Solebao Technology Co., Ltd., Beijing, China) and cultured under anaerobic conditions at 37 °C for 48 h. Single colonies exhibiting calcium-soluble rings were selected for three-zone streaking to isolate the purified strain.

### 2.2. Morphological Identification and Physicochemical Analysis of LAB

The suspected LAB was spread on an MRS solid agar plate and its colony morphology was observed. Single colonies of the suspected LAB were picked, immobilized on sterile slides, stained, and examined under a microscope for bacterial morphology [13]. Subsequently, the strains underwent detection through Gram staining and a catalase assay. For the catalase assay, add 0.5 mL of culture solution from each suspected LAB onto a glass slide, and add 5 drops of 3% H_2_O_2_ solution. Observe for bubble formation within 30 s. Strains showing bubble formation are recorded as positive; those without are recorded as negative. The isolated strains showing a purple microscopic appearance with negative catalase activity and no spores were numbered and stored at −80 °C for future reference.

### 2.3. Identification of Bacterial Strains by 16S rDNA

Genetic DNA extraction was performed using the bacterial genomic DNA extraction kit (Sangon Biotech Co., Ltd., Shanghai, China) to obtain genomic DNA [4] from isolated strains. The extracted DNA underwent 16S region amplification using primers 27F (5′-AGAGTT TGATCCTGGCTCAG-3′) and 1492R (5′-GGTTACCTTGTTACGACTT-3′). After confirming the PCR product’s purity through 1% agarose gel electrophoresis, the samples were sent to Sangon Biotech Co., Ltd. for gene sequencing. The sequence results were analyzed using the basic local alignment search tool (BLAST, https://blast.ncbi.nlm.nih.gov/Blast.cgi, accessed on 15 November 2022) in GenBank to determine the species identity of the isolated strains.

### 2.4. Screening of High-Yield Protease-LAB

The isolated strain was activated in MRS broth medium (Beijing Land Bridge Technology Co., Ltd., Beijing, China) (37 °C, anaerobic culture for 24 h). After two passages, a fermentation broth with a bacterial concentration of (8.00 ± 0.5) log CFU/mL was prepared. The broth underwent centrifugation (524r, Eppendorf (Shanghai) International Trade Co., Ltd., Shanghai, China) (4 °C, 4500× *g*, 10 min) and filtration through a 0.22 μm microporous filter membrane (Labshark, Changdu, China). A 35 μL filtrate was applied onto a proteinase screening plate (10% skim milk powder (*w*/*v*) (Feihe, Qiqihar, China), 2% agar (*w*/*v*) (Shigouyi, Nanjing, China), and 1000 mL distilled water) using a 6 mm diameter filter paper pad. The plate was cultured at 37 °C for 48 h, after which the diameter of the protease hydrolysis zone was measured using a digital vernier caliper.

A 2 mL bacterial suspension with a concentration of (8.00 ± 0.5) log CFU/mL was inoculated into MRS liquid medium and incubated at 37 °C for 48 h. The mixture was centrifuged (4 °C, 4500× *g*, 10 min) to obtain the supernatant. Proteinase activity was measured using the Folken-phenol method as described by Sun et al. [14], with bovine serum albumin used as the standard. The enzyme activity was expressed in U/mL.

### 2.5. Physicochemical Properties of High-Yield Protease LAB

#### 2.5.1. Viable Counting Method of LAB and Preparation of Bacterial Suspension

The viable counting of LAB was conducted by plate counting method, and reference was made to Hu et al. [15]. After three passages of the bacterial culture, centrifuge (4500× *g*, 10 min) and discard the supernatant. The bacterial cells were washed three times with sterile physiological saline solution under the same centrifugation conditions. A bacterial suspension with a concentration of (8.00 ± 0.5) log CFU/mL was prepared by sterile physiological saline, and stored for later use.

#### 2.5.2. Determination of Acid Production Capacity of LAB

The bacterial suspension was used at 5% (*v*/*v*) to inoculate MRS broth culture medium and cultured anaerobically at 37 °C for 24 h. The pH value of the fermentation broth was measured to determine the acid production capacity of each strain.

#### 2.5.3. Determination of LAB Hydrolyzing CGM

The bacterial suspension was used at 5.0% (*v*/*v*) to inoculate the corn protein liquid culture medium (10% *w*/*v* CGM; 4% *w*/*v* glucose; and 100 mL distilled water), and cultured at a constant temperature of 37 °C for 24 h. The soluble protein concentration was determined according to the Folin-phenol method using bovine serum albumin as the standard [16].

#### 2.5.4. Extracellular Tolerance of LAB to Gastric Juice and Bile Salts

Following the artificial gastric digestion test method by Utama et al. [17], 100 μL bacterial suspensions were transferred into 10 mL artificial gastric fluid (purchased from ZCIBIO Technology Co., Ltd., Shanghai, China) and 10 mL artificial bile (purchased from ZCIBIO Technology Co., Ltd., Shanghai, China), respectively. The cultures were incubated at 37 °C for 3 h. LAB were counted on plates before and after incubation to calculate survival rates.

### 2.6. Process Parameters of LAB-Fermented CPH

The fermentation process was carried out according to the method of Cong et al. [18]. The ratio of *Lc. rhamnosus* (ZYN-71) to *L. fermentum* (ZYN-76) was 3:1, with an inoculation volume of 4% (*v*/*v*). The amount of fructose–glucose syrup added was 5% (*v*/*v*), and the concentration of CPH was 20.5% (*w*/*v*). The fermentation temperature was 39.5 °C, and the fermentation time was 24 h.

### 2.7. Determination of Antioxidant Activity of LAB-Fermented CPH

The determination methods for DPPH and ·OH radical scavenging capacity, as well as Fe^2+^ chelation capacity, were based on the method by Cong et al. [11]. The O_2_^−^ scavenging rate measurement followed the protocol by Fu et al. [19], with the following procedure: Add Tris-HCl buffer (Thermo Fisher Scientific, Beijing, China) (0.5 mol/L, pH 8.2, 2.8 mL) and pyrogallol solution (Huasheng, Tianjin, China) (25 mM, 0.1 mL) to 0.1 mL of the sample, respectively, and mix well. Measure the absorbance at 320 nm, once every 30 s, for a total of 11 readings. Plot the absorbance values and calculate the slope, denoted as K_samples_, with distilled water serving as the blank control (K_blank_). Calculate the O_2_^−^·scavenging rate of the sample according to the following formula.

Superoxide anion clearance rate (%) = (K_blank_ − K_sample_)/K_sample_ × 100%

### 2.8. Determination of Processing Characteristics of LAB-Fermented CPH

#### 2.8.1. Preparation of CPH

The samples were lyophilized in a freeze dryer (Revo^®^ Pro, Millrock Technology, Inc., New York, NY, USA). The initial drying was performed at −40 °C for 43 h, followed by a secondary drying at 20 °C for 3 h. The resulting powder was dried at room temperature for one week and stored for later use.

#### 2.8.2. Determination of Solubility

The solubility of the samples was determined using the method of Tran et al. [20]. Fermented samples were prepared with phosphate buffered saline (PBS) buffer solutions (Thermo Fisher Scientific, Beijing, China) at different pH values (2.0, 3.0, 4.0, 5.0, 6.0, 7.0, and 8.0) to achieve varying pH levels (2.0, 3.0, 4.0, 5.0, 6.0, 7.0, and 8.0) (protein concentration: 4 mg/mL). After thorough mixing, the solutions were stored overnight in a 4 °C refrigerator. Following centrifugation at 8000× *g* for 20 min, the supernatant was collected and analyzed for protein content to calculate the sample’s solubility.

Solubility (%) = protein content in the supernatant of the sample/total protein content in the sample × 100%

#### 2.8.3. Determination of Water- and Oil-Holding Properties

The water-holding and oil-holding properties of the samples were measured using the method of Ji et al. [21]. The dried centrifuge tubes were weighed and denoted as W_1_. A 0.2 g protein sample was added and labeled as W_0_. Five mL distilled water was added to the centrifuge tube, mixed well, and centrifuged (5000× *g*, 30 min). After discarding the supernatant, the tubes were reweighed and labeled as W_2_. The water-holding property of the samples was calculated accordingly.

Water-holding capacity (g_water_/g_protein_) = (W_2_ − W_1_)/W_0_ × 100%

The protein sample (0.2 g, W_0_) was added in a dry centrifuge tube. Then, 5 mL of refined soybean oil was added and marked as V_1_. It was mixed well and centrifuged at 5000× *g* for 30 min. The volume of the supernatant was marked as V_2_. The oil retention of the sample was calculated.

Oil-holding capacity (mL_oil_/g_protein_) = (V_2_ − V_1_)/W_0_ × 100%

#### 2.8.4. Measurement of Foaming and Foam Stability

The foaming and foam stability of the fermentation product were measured using the method of Hu et al. [22]. Protein samples were prepared as a 1 mg/mL solution using 0.05 mol/L PBS buffer (pH 7.0). A 100 mL protein sample was placed in a high-speed tissue homogenizer (XHF-DY, Scientific, Ningbo, China) and stirred at 12,000 r/min for 1 min. The initial solution volume was recorded as V_0_, the foam position at stirring completion as V_L_, and the foam position after 30 min of static mixing as V_30min_. The foaming and foam stability were calculated.

Bubbling percentage (%) = V_L_/V_0_ × 100%

Foam stability (%) = V_30min_/V_0_ × 100%

#### 2.8.5. Determination of Emulsification and Emulsion Stability

The emulsification and emulsion stability of the samples were determined using the method of Hu et al. [22]. The protein sample solution was prepared by dissolving the sample in 0.05 mol/L PBS buffer (pH 7.0) at a concentration of 1 mg/mL. The sample solution was mixed with refined soybean oil and stirred at 12,000 r/min for 1 min. At the end of stirring and after 10 min of standing, 50 μL of emulsion was collected from the bottom of the beaker and transferred to a centrifuge tube. Subsequently, 5 mL of 1 mg/mL sodium dodecyl sulfate (SDS) solution was added and thoroughly mixed. The absorbance at 500 nm was measured to calculate the emulsification and emulsion stability of the fermented product.

Emulsibility (%) = 2 × T × A_0_ × dilution factor/C × (1 − ∮) × 10,000 × 100%

Emulsion stability (%) = A_10_/A_0_ × 100%

C—The concentration of the protein sample solution before emulsification (g/mL).

T—2.303.

A_0_—Absorbance at zero moment.

A_10_—The absorbance value of the emulsion after 10 min of standing.

∮—The volume fraction of oil in the solution is 0.25.

### 2.9. Determination of Volatile Substances in LAB-Fermented CPH

The volatile substances in the samples were determined by reference to the method of He et al. [23]. One gram of fermented CPH sample was taken into a 20 mL headspace flask, and headspace microextraction (45 °C, 15 min) was performed. Subsequently, desorption was carried out at 250 °C for 5 min before injection.

The volatile compounds were analyzed using a triple quadrupole GC-MS (TSQ^TM^ 9610, Thermo Fisher Scientific, Inc., Waltham, MA, USA). The GC conditions were as follows: chromatographic column: DB-5MS (30 m × 0.25 mm × 0.25 μm); column temperature: initial 40 °C maintained for 2 min, then increased at 5 °C/min to 280 °C and held for 30 min; injection port temperature: 250 °C; carrier gas flow rate: 1.0 mL/min; split ratio: no split; MS conditions: ion source temperature: 300 °C; transmission line temperature: 280 °C; and scan mode: full scan 40–550. Mass spectrometry was performed using 70 eV electron energy in the 33–400 *m*/*z* range, with only components showing a match degree greater than 80 analyzed and retention indices (RI) calculated. The relative odor activity values (ROAV) were used to determine the contribution of each volatile component to the sample’s odor profile, denoted as [24].

ROAV = (C_i_/C_max_) × (T_max_/T_i_) × 100%

C_i_—The concentration of the compound in the sample.

C_max_—The concentration of the highest concentration of all compounds in the sample.

T_max_—The olfactory threshold corresponding to the highest concentration of the compound.

T_i_—The olfactory threshold of the target compound.

### 2.10. Determination of Amino Acid Content in LAB-Fermented CPH

The amino acid was determined using the method of Li et al. [25]. The assay was performed using an amino acid fully automatic analyzer (Biochrom 30+, Harvard Bioscience Company, Cambridge, UK).

### 2.11. Data Processing

All experiments were conducted at least three times, with data results expressed as mean values ± standard deviation. Data analysis and charting were performed using Origin (OriginLab, Northampton, MA, USA), while significance tests were conducted with IBM SPSS Statistics 27 (IBM Corporation, Armonk, NY, USA). Duncan’s multiple range test was employed, where *p* < 0.05 indicated statistically significant differences. The homologous alignment of selected bacterial strain sequences was performed using the GenBank database (https://www.ncbi.nlm.nih.gov/, accessed on 15 November 2022).

## 3. Results and Analysis

### 3.1. Screening of LAB

**Figure 1:**
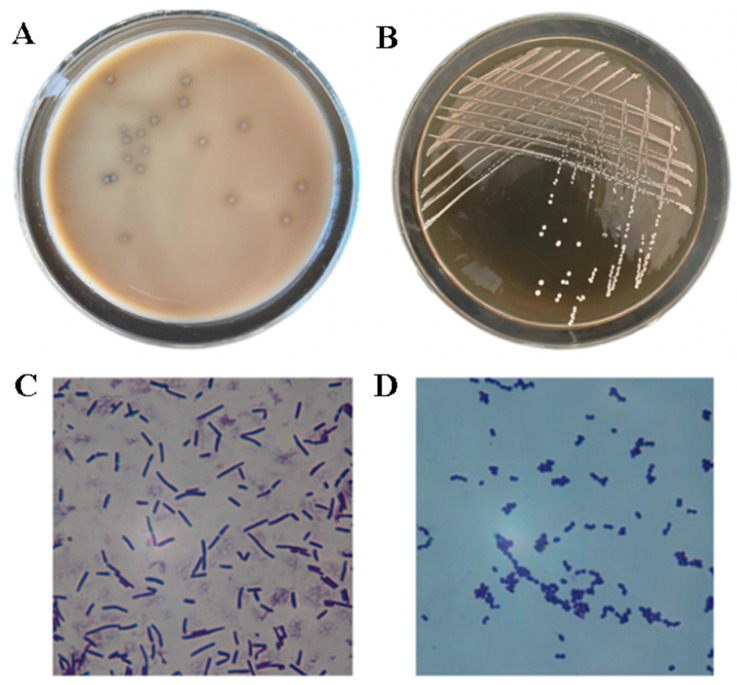
Morphological diagram of isolated strains. Note: (**A**,**B**) colony morphology of isolated strains; (**C**,**D**) morphology of isolated strains (original magnification, ×1000).

The cheese-ripening bacterial suspension was evenly spread on a 2% CaCO_3_ MRS solid medium. After 48 h of cultivation, distinct transparent halos formed around the colonies (Figure 1A). *Lactobacilli* are characterized by their ability to secrete lactic acid that dissolves CaCO_3_, leading to the preliminary identification of calcium-dissolving colonies as suspected LAB strains. These suspected LAB colonies exhibited partial whitening, opaque appearance, neat margins, and smooth, flattened circular shapes. Some colonies showed irregular white translucent forms with central circular protrusions. The suspected LAB strains were isolated using the three-zone streak technique (Figure 1B). Microscopic examination revealed short rod-shaped or spherical bacterial bodies with purple coloration, exhibiting typical Gram-positive characteristics (Figure 1C,D). Hydrogen peroxide enzyme tests demonstrated bubble formation in all tested strains, yielding negative results. These tests confirmed that the isolated strains possess characteristic LAB traits and were suspected LAB.

### 3.2. The Extracellular Protease Activity of Suspected LAB

**Figure 2:**
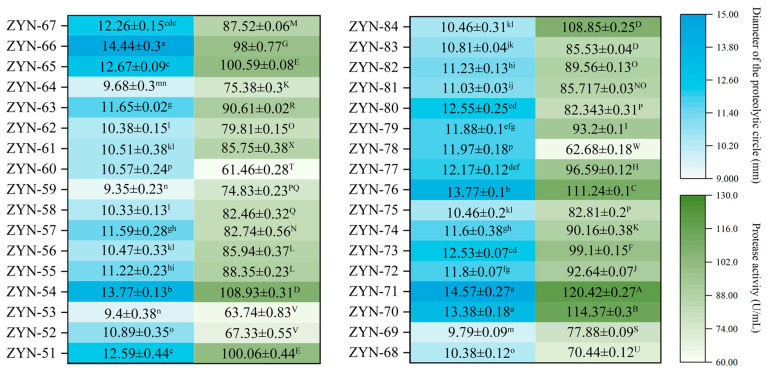
The diameter of the proteolytic circle of the isolated strain and its protease activity. Note: Different letters in each column indicate significant (*p* < 0.05) differences.

Through purification and isolation, 34 suspected LAB strains were obtained. Initial screening was conducted using protease-sensitive plates to evaluate their proteolytic zone sizes and extracellular protease activity (Figure 2). After 48 h cultivation, 34 isolate strains produced proteolytic zones ranging from 9 to 15 mm in diameter. Notably, strain ZYN-66 (14.44 mm) and ZYN-71 (14.57 mm) exhibited zones exceeding 14 mm. The protease activity ranged between 60 and 121 U/mL, with eight strains surpassing 100 U/mL and three strains (ZYN-70, ZYN-71, and ZYN-76) exceeding 110 U/mL. Ma et al. [26] conducted a study on the use of the high protease production LAB for the fermentation of soybean meal as animal feed. It found that 22 strains showed protease activity greater than 20.00 U/mL after initial screening. This demonstrates that strains with higher protease production can be effectively identified from fermented products containing elevated protein content.

### 3.3. DNA Extraction and PCR Amplification of Suspected LAB

**Figure 3:**
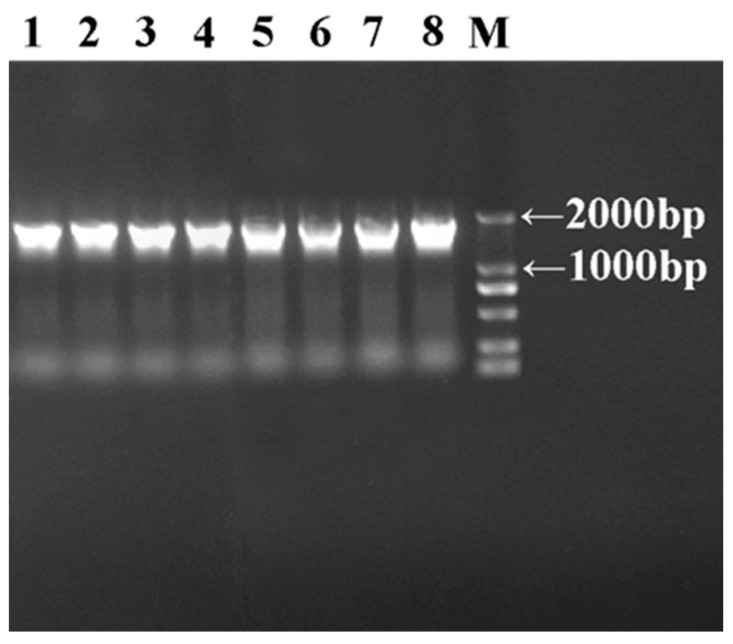
Electrophoresis diagram of PCR products of eight isolated strains. Note: M: marker; 1: ZYN-51; 2: ZYN-54; 3: ZYN-65; 4: ZYN-66; 5: ZYN-70; 6: ZYN-71; 7: ZYN-76; and 8: ZYN-84.

Whole genome DNA was extracted from eight isolated strains (with protease activity exceeding 100 U/mL) and subjected to PCR amplification in vitro (Figure 3). The marker bands were complete, with all strains displaying a clear and distinct band between 1000 bp and 2000 bp, which served as a reliable identification marker. Through BLAST (https://blast.ncbi.nlm.nih.gov/Blast.cgi) homology alignment against the GenBank database, the sequencing results identified eight strains as *Lactiplantibacillus* and *Limosilactobacillus* within the Firmicutes phylum (Table 1). Specifically, five strains belonged to *Lactiplantibacillus plantarum* (ZYN-51, ZYN-65, ZYN-66, ZYN-70, ZYN-84), one strain was *Lacticaseibacillus paracasei* (ZYN-54), one strain was *Lc. rhamnosus* (ZYN-71), and one strain was *L. fermentum* (ZYN-76).

### 3.4. Characteristic Analysis of High-Yield Protease-LAB

#### 3.4.1. The Ability of LAB to Produce Acid and Hydrolyze CGM

**Figure 4:**
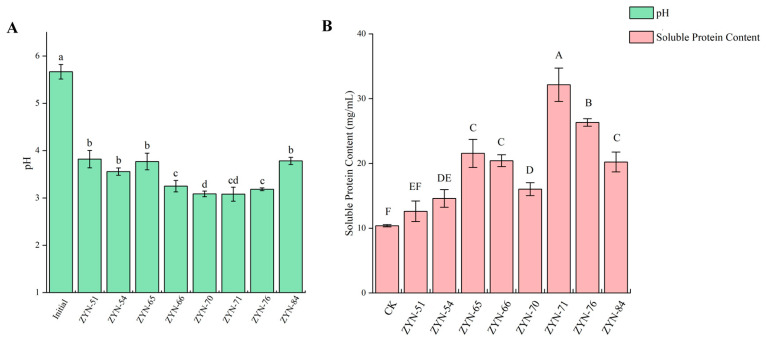
The ability of LAB to produce acid (**A**) and hydrolyze CGM (**B**). Note: CK is non-inoculated. The data are mean ± standard deviation, and different letters in each column indicate significant (*p* < 0.05) differences.

After 24 h of cultivation, the acid-producing capacity of eight isolated strains showed significant differences (*p* < 0.05) (Figure 4A). The initial pH value in the culture medium was 5.80. After fermentation, the pH value of eight isolated strains ranged between 3.00 and 3.90. Among them, strains ZYN-66 (3.18), ZYN-71 (3.01), ZYN-70 (3.03), and ZYN-76 (3.15) exhibited stronger acid-producing capability, while the pH value of the other strains was above 3.60. Boeck et al. [24] studied LAB-fermented legume protein isolate (LPI) as a yogurt substitute, and found that three LABs had a high acid-producing capacity, reducing the pH value of the fermented liquid from 6.92 to 3.60–4.80. This indicates that the production capabilities of LAB selected from different fermentation substrates show significant variations, likely due to differences in their adaptability to fermentation environments and nutrient utilization capacities.

CGM contains a lot of zein, and its modification test was conducted using eight isolated strains. After 24 h cultivation, there were significant differences in the CGM hydrolysis capacity of eight isolated strains (*p* < 0.05) (Figure 4B). The soluble protein content in the fermentation broth of eight isolated strains ranged from 12 to 33 mg/mL after fermentation. Strains ZYN-71 (32.14 mg/mL) and ZYN-76 (26.32 mg/mL) showed a strong ability to hydrolyze CGM, with soluble protein content increases of 209.63% and 153.56%, respectively. Wronkowska et al. [27] studied the production of biscuits through LAB fermentation of buckwheat flour. They found that after specific LAB fermentation of buckwheat flour, the soluble protein content could be increased by at least 14.4%. It is indicated that LAB can hydrolyze macromolecular proteins, and effectively enhance soluble protein content in raw materials [28]. The elevated protein content may be attributed to variations in protease composition [29].

#### 3.4.2. Resistance to Artificial Bile and Gastric Juice

The ability of LAB to enter the intestinal tract and exert probiotic effects primarily depends on their successful passage through the human digestive system. After being cultured in gastric juice (pH 2.0) for 3 h, eight isolated strains exhibited varying tolerance levels (*p* < 0.05), with survival rates ranging from 8% to 64% (Table 2). Strains ZYN-66 (55.65%), ZYN-70 (55.41%), ZYN-71 (63.77%), and ZYN-76 (62.38%) showed better gastric tolerance, achieving over 50% survival. When cultured in artificial bile (0.3% concentration) for 3 h, the survival rates of eight strains were maintained between 4% and 21%. Strains ZYN-66 (19.54%), ZYN-70 (16.83%), ZYN-71 (20.43%), and ZYN-76 (19.41%) demonstrated superior bile tolerance with survival rates exceeding 15% (*p* < 0.05). Pamela et al. [30] investigated the probiotic potential of two natural LAB (*Leuconostoc pseudomesenteroides* and *Weissella confusa*). They found that two strains showed survival rates reaching 54.15–57.04% after 4 h of cultivation in simulated gastric fluid (pH 2.0), indicating their suitability for intestinal colonization.

#### 3.4.3. Principal Component Analysis (PCA)

**Figure 5:**
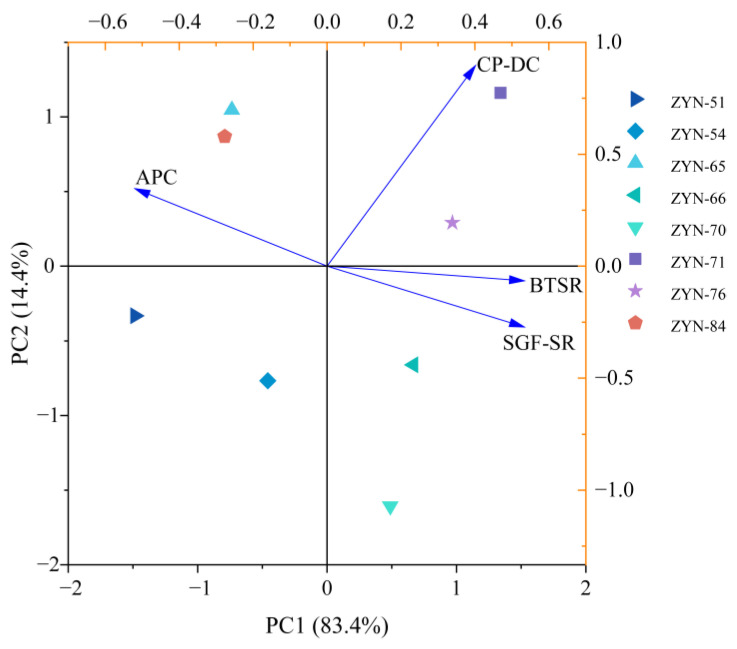
Screening LAB principal component analysis. Note: APC is acid production capacity; CP-DC is hydrolysis capacity; BTSR is bile tolerance survival rate; and SGF-SR is simulated gastric fluid survival rate.

Principal component analysis (PCA) was conducted on eight LAB strains with screening parameters (Figure 5). The contribution rate of Principal Component 1 (PC 1) was 83.4%, Principal Component 2 (PC 2) was 14.4%, and the cumulative contribution rate was 97.8%. This indicated that the model accurately reflected the relationship between bacterial strains and screening conditions. Eight LAB strains were distributed across four quadrants. The strains located in Quadrant III (ZYN-51, ZYN-54) and Quadrant IV (ZYN-66, ZYN-70) showed low correlations with each of the screening conditions. Strains ZYN-65 and ZYN-84 located in Quadrant II (upper left) exhibited strong correlation with acid production capacity. Strains ZYN-71 and ZYN-76 located in Quadrant I (upper right) demonstrated significant correlations with CGM hydrolysis capability, artificial gastric fluid survival rate, and bile survival rate. Consequently, strains ZYN-71 and ZYN-76 were selected as fermentation strains for CPH bio-fermentation modification.

### 3.5. Effect of LAB Fermentation on Antioxidant Activity of CPH

**Figure 6:**
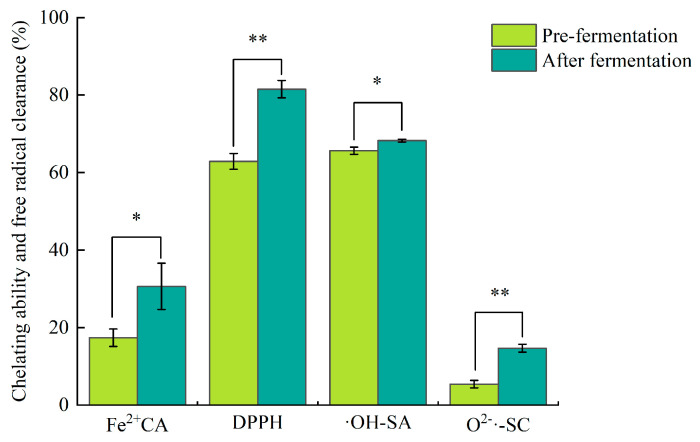
Effects of LAB on antioxidant activity of CPH before and after fermentation. Note: All of them were in the condition of protein content of 1 mg/mL. Different letters in each column indicate significant (* *p* < 0.05, ** *p* < 0.01) differences.

The antioxidant activity of CPH fermented by LAB increased to varying degrees before and after fermentation (*p* < 0.05) (Figure 6). After fermentation, the O^2−^ radical scavenging capacity demonstrated the most substantial enhancement at 63.37% (IC_50_ = 12.851 mg/mL, measured as 0.1 mg/mL Vc). The Fe^2+^ chelation capacity increased by 43.27% (IC_50_ = 1.856 mg/mL, measured as 0.1 mg/mL Vc), while the DPPH free radical scavenging capacity increased by 22.85% (IC_50_ = 0.227 mg/mL, measured as 0.1 mg/mL Vc). The ·OH radical scavenging capacity showed the smallest improvement at 3.82% (IC_50_ = 0.190 mg/mL, measured as 0.1 mg/mL Vc). The enhancement of antioxidant capacity is mainly attributed to the oligopeptides and free amino acids produced by LAB during the fermentation of CPH [31]. It has been reported that low-molecular-weight peptides with specific amino acid sequences and some free amino acids have strong antioxidant capabilities. Additionally, the extracellular proteases and metabolic products (e.g., lactic acid) by LAB can disrupt the tertiary structure of proteins, breaking disulfide bonds, hydrogen bonds, and other covalent bonds, exposing functional groups with antioxidant capabilities, and thereby enhancing the free radical scavenging ability or metal ion chelation ability [32]. Furthermore, LAB also possess certain antioxidant capabilities. The LAB screened by Cong et al. from blueberry peels all have certain antioxidant abilities. Among them, *Lactiplantibacillus planturum* ZYN-0221 and *Lacticaseibacillus rhamnosus* ZYN-0417 exhibit strong antioxidant capabilities [12]. Mechanisms employed by LAB to remove oxygen radicals may rely on enzymes (superoxide dismutase (SOD), catalase, NADH oxidase, and NADH peroxidase), extracellular polysaccharides, or non-enzyme agents (Mn^2+^, ascorbate, tocopherols, and glutathione) [33].

### 3.6. Effects of LAB Fermentation on Processing Characteristics of CPH

**Figure 7:**
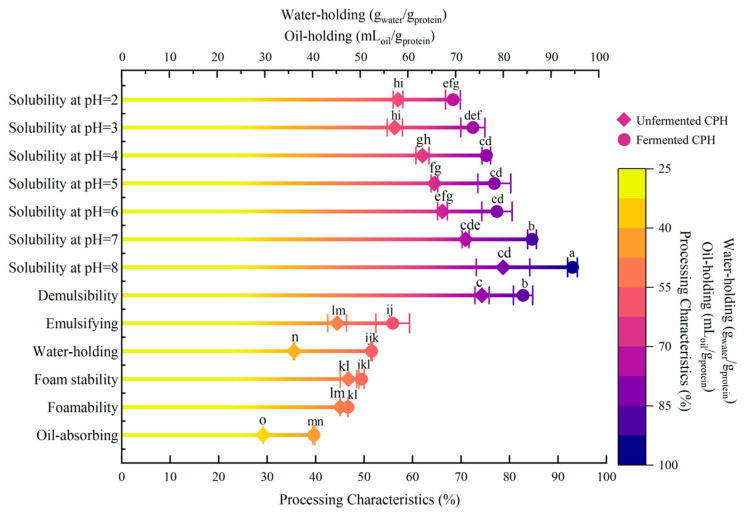
Effect of LAB on the processing characteristics of CPH before and after fermentation. Note: The data in this figure are mean ± standard deviation, and different letters in each column indicate significant (*p* < 0.05) differences.

The processing properties determine the application scope, product stability, and flavor characteristics of CPH. Under different pH values (2.0–8.0), the solubility of fermented CPH increased significantly, ranging from 11.0% to 16.5% (Figure 7). Notably, the maximum increase occurred at pH 3.0 (16.1%). The foaming performance improved by 1.61%, while foam stability was enhanced by 6.85%. After fermentation, emulsification increased by 11.47% (*p* > 0.05), with emulsion stability rose by 8.48%. Water retention and oil retention increased by 16.02% and 10.5%, respectively. These results demonstrate that proteases and organic acids [34] produced by LAB effectively disrupt the spatial conformation of proteins [35] in CPH. They cause structural loosening, weakening intermolecular forces [36], and the breaking of some chemical bonds, thereby providing favorable processing characteristics.

### 3.7. Changes in Amino Acid Content of LAB-Fermented CPH

A total of 16 distinct amino acids were identified in CPH before and after fermentation (Table 3). LAB fermentation resulted in a general decrease in amino acid content, with total amino acid levels dropping by 18.48 g/100 g. The most significant reduction occurred in bitter-tasting amino acids, decreasing by 7.31 g/100 g. This indicated that during LAB fermentation, the utilization rate of free amino acids in CPH was higher than the hydrolysis rate. Furthermore, it changed the amino acid composition of CPH [37], and reduced the proportion of bitter amino acids [38].

### 3.8. Effects of LAB Fermentation on the Volatile Substances of CPH

**Figure 8:**
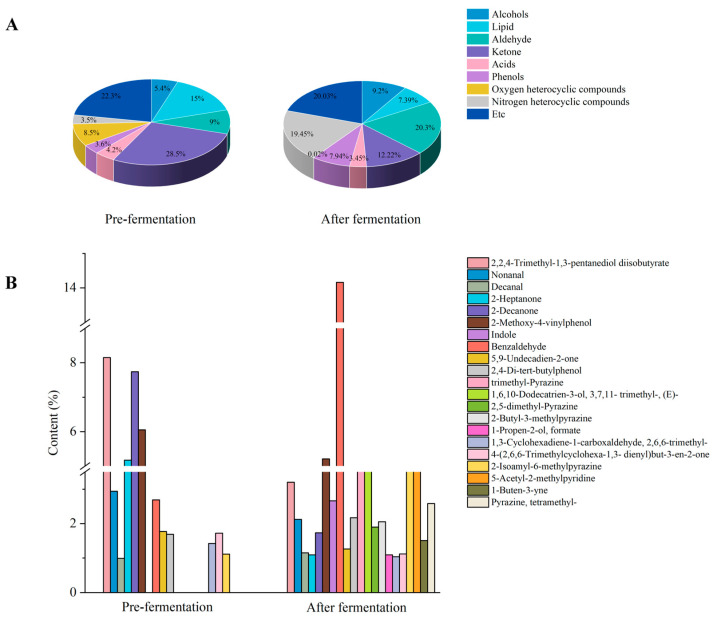
Key volatile components and their types and contents in CPH before and after LAB fermentation. Note: (**A**) shows the types and contents of volatile components of CPH before and after fermentation; (**B**) shows the key volatile components in the CPH before and after fermentation (ROAV ≥ 1 after fermentation is the main reference).

Through headspace solid-phase microextraction gas chromatography-mass spectrometry (HS-SPME-GC-MS) analysis, 109 volatile flavor compounds were identified in fermented CPH before and after fermentation, primarily including alcohols, esters, aldehydes, phenols, acids, alkanes, terpenes, oxygen-containing heterocyclic compounds, nitrogen-containing heterocyclic compounds, and other types of compounds (Figure 8A). The ketones (28.5%) were the dominant component before fermentation, while aldehydes (20.2%) and nitrogen-containing heterocyclic compounds (19.4%) became predominant after fermentation. Specifically, the relative content of alcohols increased by 3.8%, aldehydes by 11.3%, and nitrogen-containing heterocyclic compounds by 15.9%. These changes imparted distinct nutty and baked aromas to the fermented CPH [39].

There were 15 key volatile components (ROAV ≥ 1) in CPH before fermentation, primarily including 2,2,4-trimethyl-1,3-pentanediol diisobutyrate (8.15%), methyl octyl ketone (7.74%), and 4-vinyl-2-methoxyphenol (6.05%) (ROAV ≥ 6) (Figure 8B). Seven volatile components (0.1 ≤ ROAV ≤ 1) contribute to overall aroma modification. CPH after fermentation showed 17 key volatile components, mainly including benzaldehyde (14.16%), 4-vinyl-2-methoxyphenol (5.21%), and 2-isopentyl-6-methylpyridine (4.03) (ROAV ≥ 4), with six components modifying the overall aroma profile. During LAB fermentation, aromatic amino acids (e.g., Phe) are converted into benzaldehyde and phenylpyruvic acid through transaminase action. The phenylpyruvic acid further metabolizes into phenylacetaldehyde [40]. The benzaldehyde and phenylacetaldehyde impart pleasant aromas [41]. Notably, odor components such as diethyl disulfide and indole were significantly reduced or eliminated after fermentation. This demonstrated that LAB fermentation comprehensively enhanced the aroma profile of CPH.

## 4. Conclusions

The traditional biological method was used to isolate 34 suspected LAB strains from cheese, with eight strains demonstrating strong protease secretion capabilities. Through further screening for acid tolerance and artificial bile and gastric fluid adaptability, as well as CGM hydrolysis capacity, strains ZYN-71 and ZYN-76 were identified as suitable for the fermentation of CPH. When co-fermented with the mixed bacterial strain, the CPH showed significant improvements in antioxidant activity and processing characteristics, with a substantial reduction in bitter-tasting amino acids. Notably, aroma compounds increased while off-flavors nearly disappeared before and after fermentation. The results indicated that, *Lc. rhamnosus* ZYN-71 and *L. fermentum* ZYN-76 from cheese exhibited excellent fermentation capabilities for CPH. After fermentation, the properties and flavor of CPH have been significantly improved. This is expected to solve the problems existing in the practical application of CPH.

## Figures and Tables

**Table 1:** Identification results of 16S rDNA of eight isolated strains.

Generic Name	Specific Name	Strain Number
*Lactiplantibacillus*	*plantarum*	ZYN-51, ZYN-65, ZYN-66, ZYN-70, ZYN-84
*Limosilactobacillus*	*fermentum*	ZYN-76
*Lacticaseibacillus*	*paracasei*	ZYN-54
	*rhamnosus*	ZYN-71

**Table 2:** The tolerance of LAB to artificial bile and gastric juice.

Serial Number	Survival Rate in Artificial Bile (%)	Survival Rate in Artificial Gastric Juice (%)
ZYN-51	4.03 ± 0.54 ^f^	8.03 ± 1.54 ^g^
ZYN-54	13.3 ± 0.11 ^d^	32.95 ± 3.11 ^d^
ZYN-65	10.22 ± 0.92 ^e^	13.45 ± 2.90 ^f^
ZYN-66	19.54 ± 0.24 ^b^	55.65 ± 3.42 ^c^
ZYN-70	16.83 ± 0.05 ^c^	55.41 ± 3.25 ^c^
ZYN-71	20.43 ± 0.80 ^a^	63.77 ± 2.80 ^a^
ZYN-76	19.41 ± 0.05 ^b^	62.38 ± 3.85 ^b^
ZYN-84	10.87 ± 0.28 ^e^	15.49 ± 6.28 ^e^

Note: The data in this table are mean ± standard deviation, and different letters in each column indicate significant (*p* < 0.05) differences.

**Table 3:** The content of amino acids in CPH before and after LAB fermentation.

Taste	Amino Acid	CPH (g/100 g)	CPH After Fermentation (g/100 g)	Amino Acid	CPH (g/100 g)	CPH After Fermentation (g/100 g)
Palatable taste	Asp	4.57	3.61	Glu	17.66	13.65
Amount		22.23	17.26			
Sweet taste	Gly	1.89	1.48	Ser	4.02	3.00
	Thr	2.71	2.03	Ala	6.23	4.40
	Pro	5.62	4.88			
Amount		20.05	15.79			
Bitter taste	Lys	0.99	0.97	Tyr	3.70	2.31
	His	1.40	1.28	Val	3.42	2.61
	Met	1.79	1.29	Phe	4.46	3.38
	Arg	1.94	1.30	Leu	13.16	9.60
	Ile	2.92	2.21			
Amount		33.79	24.95			
Hydrophobic amino acid	37.61	28.37				
Total amino acids	76.48	58				

## Data Availability

The original contributions presented in the study are included in the article; further inquiries can be directed to the corresponding author.
